# Virus-Specific T Cell Therapies for HIV: Lessons Learned From Hematopoietic Stem Cell Transplantation

**DOI:** 10.3389/fcimb.2020.00298

**Published:** 2020-07-07

**Authors:** Ping-Hsien Lee, Michael D. Keller, Patrick J. Hanley, Catherine M. Bollard

**Affiliations:** ^1^Center for Cancer and Immunology Research, Children's National Hospital, Washington, DC, United States; ^2^Division of Allergy & Immunology, Children's National Hospital, Washington, DC, United States; ^3^Division of Blood and Marrow Transplantation, Children's National Hospital, Washington, DC, United States; ^4^GW Cancer Center, The George Washington University, Washington, DC, United States

**Keywords:** HIV, T cells, immunotherapy, hematopoietic stem cell transplantation, latent reservoir

## Abstract

Human immunodeficiency virus (HIV) has caused millions of deaths and continues to threaten the health of millions of people worldwide. Despite anti-retroviral therapy (ART) substantially alleviating severity and limiting transmission, HIV has not been eradicated and its persistence can lead to other health concerns such as cancer. The only two cases of HIV cure to date are HIV^+^ cancer patients receiving an allogeneic hematopoietic stem cell transplantation (allo-HSCT) from a donor with the CCR5 Δ32 mutation. While this approach has not led to such success in other patients and is not applicable to HIV^+^ individuals without cancer, the encouraging results may point toward a breakthrough in developing a cure strategy for HIV. Adoptive transfer of virus-specific T cells (VSTs) post HSCT has been effectively used to treat and prevent reactivation of latent viral infections such as cytomegalovirus (CMV) and Epstein-Barr virus (EBV), making VSTs an attractive therapeutic to control HIV rebound. Here we will discuss the potential of using adoptive T cell therapies in combination with other treatments such as HSCT and latency reversing agents (LRAs) to achieve a functional cure for HIV.

## Introduction

According to the latest UNAIDS statistics, HIV impacted an estimated 37.9 million individuals worldwide, and resulted in over 770,000 deaths by the end of 2018. Unfortunately, a cure for the immunity-demolishing virus is still lacking. T cells play a key role in the immune response against viral infections, including HIV. Anti-HIV CD8^+^ T cell responses can be induced upon infection and are associated with a transient reduction in viral load (Walker et al., 1987; Koup et al., 1994). In most cases without any medical intervention, the anti-viral cellular responses fail to clear the virus, leading to a progressive loss of CD4^+^ T cells and eventually a state with a severely impaired immune system known as acquired immunodeficiency syndrome (AIDS). A combination of drugs targeting different stages of HIV life cycle in anti-retroviral therapy (ART) has been used to treat HIV infection, rendering the virus undetectable and preventing transmission (Arts and Hazuda, 2012). Nevertheless, ART requires life-long adherence to medication and does not eradicate the virus (Wong et al., 1997; Chun et al., 1999). Even with active viral replication being halted by ART, the quiescent immune cells—especially memory CD4^+^ T cells—harboring replication-competent provirus can persist and constitute the latent HIV reservoir (Chun et al., 1995; Strain et al., 2005; Archin et al., 2012a). HIV reservoir can evade the immune system due to the lack of viral antigen expression and can cause viral rebound after the cessation of ART, making them the major barrier to an HIV cure (Sengupta and Siliciano, 2018).

Both individuals cured of HIV have received HSCT with CCR5 Δ32 donor cells (Hutter et al., 2009; Gupta et al., 2019). This suggests that eradication of HIV requires a reduction in the viral reservoir by myeloablation (Henrich et al., 2013, 2014) followed by immune reconstitution with HIV-resistant cells to prevent further infection. However, the emergence of viral rebound after ART interruption in other patients undergoing similar procedures (Hutter, 2014; The Lancet, 2019) indicates that additional therapeutics are needed to help control viral spread from the lasting latent reservoir. The ability of adoptive transfer of *ex vivo*-grown, virus-specific T cells to treat and prevent viral infections (e.g., CMV and EBV) in immunocompromised patients (Leen et al., 2006; Saglio et al., 2014) makes adoptive T cell therapy (ACT) a promising strategy to prevent HIV rebound post HSCT. Here we will focus on the clinical efforts of using ACT in treating HIV infection (Table 1) and discuss the potential of using ACT in combination with HSCT and LRA to eradicate the latent reservoir.

**Table 1:** Summary of T-cell-based therapies in HIV clinical trials.

**Strategy**	**ART status**	**Trial status (start date/completion date)**	**Clinicaltrials.gov identifier**	**Locations**	
Autologous T cells transduced with a CD4z CAR in combination with IL-2	ART suppressed	Active and not recruiting (2001-09/N.A.)	NCT01013415 (Scholler et al., 2012)	• University of Pennsylvania, Philadelphia, Pennsylvania, United States	
Autologous T cells transduced with a bNAb-based CAR	ART suppressed	Active and recruiting (2017-10/N.A.)	NCT03240328	• Guangzhou 8th People's Hospital, Guangzhou, Guangdong, China	
Autologous CD8^+^ T cells transduced with two gag-specific TCRs	Subject to ART interruption post infusion	Completed (2009-11/2014-01)	NCT00991224	• University of Pennsylvania, Philadelphia, Pennsylvania, United States	
Autologous mitogen-expanded CD8^+^ T cells	ART suppressed	Completed (N.A./2002-06)	NCT00000756 (Lieberman et al., 1997)	• New England Medical Center/Tufts University, Boston, Massachusetts, United States	
Autologous HIV peptide-expanded CD8^+^ T cell clones	ART suppressed	Completed (1998-09/2005-04)	NCT00110578 (Chapuis et al., 2011)	• Fred Hutchinson Cancer Research Center, Seattle, Washington, United States • University of Washington, Seattle, Washington, United States	
Autologous HIV peptide-expanded multi-specific T cells	Treated with ART during acute or chronic infection	Completed (2015-04/2017-11)	NCT02208167 (Sung et al., 2018)	• University of North Carolina, Chapel Hill, North Carolina, United States	
Autologous HIV peptide-expanded multi-specific T cells	ART suppressed	Active and recruiting (2019-03/N.A.)	NCT03485963	• Whitman Walker Health Research Department, Washington, District of Columbia, United States • Children's National Health System, Washington, District of Columbia, United States • Children's National Medical Center, George Washington University Hospital and Whitman Walker Health Research Department, Washington, District of Columbia, United States	
Autologous T cells transduced with a bNAb-based CAR in combination with Chidamide	ART suppressed	Active and recruiting (2017-12/N.A.)	NCT03980691	• Guangzhou 8th People's Hospital, Guangzhou, Guangdong, China	
Autologous HIV peptide-expanded multi-specific T cells in combination with Vorinostat	ART suppressed	Active and recruiting (2017-06/N.A.)	NCT03212989	• University of North Carolina, Chapel Hill, North Carolina, United States	
Autologous CD4^+^ T cells transduced with ZFN to disrupt CCR5	Subject to ART interruption post infusion	Completed (2009-01/2013-01)	NCT00842634 (Tebas et al., 2014)	• Jacobi Medical Center, Bronx, New York, United States • University of Pennsylvania, Philadelphia, Pennsylvania, United States	
Autologous CD4^+^ T cells transduced with ZFN to disrupt CCR5	ART suppressed with one cohort being subject to treatment interruption	Completed (2009-12/2014-12)	NCT01044654	• Nine institutes in California, Connecticut, Florida, Missouri, New Mexico, New York, and Texas, United States	
Autologous CD4^+^ T cells transduced with ZFN to disrupt CCR5	Non-ART suppressed (HIV viral load between 1e3 and 1e6 copies/ml)	Completed (2010-11/2015-05)	NCT01252641	• Four institutes in California and Florida, United States	
Autologous CD4^+^ T cells transduced with C34-CXCR4	Subject to ART interruption post infusion	Completed (2017-01/2020-03)	NCT03020524	• University of Pennsylvania, Philadelphia, Pennsylvania, United States	
Autologous HSPC and CD4^+^ T cells transduced with a CCR5-targeting shRNA and C46 with or without preconditioning for transplant	ART suppressed	Completed (2013-04/2017-11)	NCT01734850	• UCLA CARE Center, Los Angeles, California, United States • Quest Clinical Research, San Francisco, California, United States	
Autologous T cells transduced with a CD4 CAR and ZFN to disrupt CCR5	Subject to ART interruption post infusion	Active and not recruiting (2019-07/N.A.)	NCT03617198	• University of Pennsylvania, Philadelphia, Pennsylvania, United States	
Autologous CD4+ T cells transduced with an *env*-targeting antisense	Subject to ART interruption post infusion	Completed (2006-01/2013-12)	NCT00295477 (Tebas et al., 2013)	• University of Pennsylvania, Philadelphia, Pennsylvania, United States	

## The Success of Using VSTs to Prevent and Treat Viral Infections Post HSCT

HSCT is considered a curative therapy for hematological malignancies and certain genetic disorders, but viral infections post HSCT are a major cause of morbidity and mortality (Boeckh et al., 2003; Myers et al., 2005; Brunstein et al., 2006). This is because the recipients are devoid of anti-viral immunity when the T cell compartment slowly recovers and immunosuppression is typically used to prevent and treat graft-vs.-host disease (GVHD). While prophylactic and therapeutic pharmacological treatments are available, they can be expensive and their efficacy is often limited by the toxicity and drug resistance (Tomblyn et al., 2009). ACT is an alternative approach showing clinical benefits for viral disease in immunodeficient transplant recipients. Adoptive transfer of donor-derived CMV-specific cytotoxic T lymphocyte (CTL) clones prevents CMV infection (Riddell et al., 1992). Infusions of unselected leukocytes or EBV-specific CTLs derived from EBV-seropositive HSCT donors effectively treated EBV-associated lymphoproliferative disease (Papadopoulos et al., 1994; Doubrovina et al., 2012). When endowed with a CD19-targeting artificial receptor, VSTs also provided antitumor activity against relapsed B cell malignancies post HSCT (Cruz et al., 2013). These findings demonstrate the potential of using antigen-specific T cells as “living drugs” to quickly reconstitute recipient's T cell responses to treat rapid-progressing and life-threatening viral complications post HSCT.

Tremendous efforts have been made to refine the manufacture of cell products, aiming at conferring durable protection without causing GVHD by reproducibly generating clinical doses of T cells with the desired specificity, long-term *in vivo* persistence, and minimal alloreactivity. While it has yet been adapted to other viruses, ACT has emerged as a safe and efficient therapeutic for combating CMV, EBV, and adenovirus infections (Cruz et al., 2010; Mui et al., 2010; Bollard, 2013; Houghtelin and Bollard, 2017; Kaeuferle et al., 2019). Traditionally, the source of VSTs is limited to the stem cell donor, which may not be a readily available VST donor source to treat immunocompromised patients suffering from devastating viral diseases. In contrast, “off-the-shelf” VSTs that have been generated from “third-party donors” with diverse HLA types and banked can be used to treat high-risk patients even if HLA matching between the third-party donor and the patient is only at a single allele. Hence, the emerging use of third-party VSTs has broadened the applicability of adoptive T cell therapy for viral infections (Haque et al., 2007; Leen et al., 2013). For these reasons, infusion of HIV-specific T cells could be a logical treatment option to restore anti-HIV immunity in infected individuals.

## From EBV, CMV, Adenovirus to HIV

ART has effectively decreased the incidence of HIV-infected individuals progressing to AIDS and the subsequent AIDS-defining illnesses, but the deaths by non-AIDS-defining cancers have been escalating (Deeken et al., 2012). The Berlin patient, the very first case of HIV cure, developed acute myeloid leukemia (AML) while on ART and received two HSCTs from an HLA-matched donor with the homozygous CCR5 Δ32 mutation (Hutter et al., 2009). He has remained devoid of detectable virus for >10 years since the discontinuation of ART (Lederman and Pike, 2017). The other case is the London patient, who received a less aggressive conditioning regimen for Hodgkin's lymphoma and has been in HIV remission without ART since September, 2017 (Gupta et al., 2019, 2020). Myeloablative conditioning not only killed cancer cells but substantially reduced the size of the HIV reservoir (Koelsch et al., 2017; Salgado et al., 2018). However, the reduction in the HIV reservoir alone appears insufficient to achieve a cure because viral rebound was observed after stopping ART in patients receiving HSCT from CCR5 wild-type donors (Henrich et al., 2013, 2014; Cummins et al., 2017).

In addition to a substantial reduction in the HIV reservoir, achieving full donor chimerism with allogeneic HIV-resistant donor cells is critical for preventing viral rebound. However, the success of the Berlin and London patients has not been reproduced in other patients transplanted with CCR5 Δ32 donors (even homozygous donors) (Hutter, 2014; The Lancet, 2019). The only possible exception is that of the Düsseldorf patient who has been in remission since the discontinuation of ART in November, 2018 as reported by the IciStem (International Collaboration to guide and investigate the potential for HIV cure by Stem Cell Transplantation) consortium (The Lancet, 2019). The failure to achieve a cure in most patients was likely due to the high post-transplant mortality and the emergence of an X4-tropic escape variant (Hutter, 2014; Kordelas et al., 2014; Duarte et al., 2015; Rothenberger et al., 2018). Nevertheless, aside from the rarity of donors with the homozygous mutant alleles (Solloch et al., 2017) and the toxicity of the allograft procedure, these studies suggest that allo-HSCT using donor cells that are resistant to HIV infection along with continuous ART could effectively reduce and maintain the size of the HIV reservoir at low levels. Despite not being curative for most patients, this approach could however open a window for adoptive anti-viral immunity to prevent viral rebound, given the success of infusing VSTs to treat EBV, CMV and adenovirus infections in HSCT recipients.

## Adoptive T-Cell Therapy for HIV

ACT is an efficient way to quickly replenish a patient's T cell responses. With advancing culture procedures and genome-editing techniques, autologous or allogeneic T cells can be expanded *ex vivo* and endowed with antigen-specificity and improved persistence and function within the tissue microenvironment. While ACT had little success with HIV treatment in the past (Koenig et al., 1995; Lieberman et al., 1997), infusion of improved T cell products along with efficient ways to reduce the viral reservoir and increase immunogenicity of the viral antigens may offer an opportunity to eradicate the virus in ART-treated patients.

### HIV-Specific Chimeric Antigen Receptors

The chimeric antigen receptor (CAR) is an artificial receptor with an extracellular antigen-binding domain connected to an intracellular signaling domain by a hinge region and a transmembrane domain. When expressed on the surface of a T cell, CAR can induce T-cell activation upon ligand binding independently of T cell receptor (TCR)-major histocompatibility complex (MHC) interactions, thereby bypassing MHC restrictions and MHC downregulation mechanisms often used by cancer cells or viruses, including HIV (Dotti et al., 2014; Wagner, 2018). Despite the recent success in treating hematological malignancies, CAR-T-based therapies are limited by obstacles such as antigen loss, poor T cell persistence, and immune-related toxicities (Sun et al., 2018; Shah and Fry, 2019). While HIV-targeting CARs showed limited success in the early clinical trials, primarily due to viral escape variants and susceptibility of CAR-T cells to HIV infection, new CAR-based therapies developed to overcome these hurdles have shown promising results in preclinical studies (reviewed in Kuhlmann et al., 2018; Wagner, 2018; Yang et al., 2018; Kim et al., 2019; Qi et al., 2020). Their clinical efficacy is being actively investigated (Table 1).

### HIV-Specific TCRs

Naturally-occurring TCRs can also recognize HIV-associated antigens in the context of MHC and therefore can be used to engineer T cell specificity. An artificial TCR specific for the HLA-A^*^02-restricted p17 *gag* epitope SLYNTVATL (SL9) was derived from a naturally-occurring TCR isolated from an HIV-infected individual and demonstrated a higher affinity to SL9 (Varela-Rohena et al., 2008). CD8^+^ T cells transduced with this supraphysiologic TCR can better control the infection with wild-type and SL9 escape variants of HIV than the original TCR *in vitro*. A phase 1 trial was initiated to test T cells transduced with these TCRs either before or during ART interruption (NCT00991224), but the results have not been reported. Engineering T cell specificity with TCRs may yield a highly efficient T cell response, but its use is limited by MHC restriction, antigen loss, and off-target toxicities including death as reported for cancer patients infused with MAGE-A3 TCR T cells (Cameron et al., 2013; Linette et al., 2013).

Another option to achieve HIV specificity is to use CTL lines or clones derived from preformed memory T cells, which exist at high frequencies in HIV-infected individuals (Hoffenbach et al., 1989). Mitogen-expanded, autologous CD8^+^ T cell lines enriched for reactivity against HIV antigens gp120, p17, p24, and Nef were infused to six HIV-infected subjects. Despite increased CD4^+^ T cell counts and decreased plasma viremia following CTL infusion, the effects were transient (2 weeks) and did not reach statistical significance (NCT00000756 Lieberman et al., 1997). In another study, autologous HIV Gag-specific CD8^+^ T cell clones obtained by limiting dilutions were infused to three ART-suppressed subjects. Infusion of CTL was accompanied by a reduction in HIV-infected CD4^+^ T cells in the peripheral blood, but the reduction was transient and the plasma HIV RNA levels were not diminished (Brodie et al., 1999). While utilizing CD8^+^ T cell lines or clones may be an efficient and low-risk way to obtain clinically relevant numbers of HIV-targeting T cells without any genetic manipulation, their antigen breadth is still limited and may even cause a selective expansion of antigen escape variants (Koenig et al., 1995). Moreover, the lack of CD4^+^ T cells in the infused cell products may result in an impaired persistence of HIV-specific CD8^+^ T cells *in vivo*. This will be discussed in more detail below.

### HIV-Targeting Multi-Specific T Cells

With the understanding that the mutation rate of HIV *in vivo* may be higher than that originally estimated (Cuevas et al., 2015), targeting multiple epitopes from different antigens simultaneously presents a logical solution to conquer immune escape by increasing T cell target breadth. It was first shown that a polyclonal HIV-specific T cell population can be obtained from HIV seropositive blood samples by three rounds of stimulation with peptide-pulsed antigen-presenting cells (APCs) (Lam et al., 2015). The peptides used for T cell stimulation were pools of ~150 15-mers spanning the most conserved regions of *gag, pol*, and *nef* across all clades of HIV. Due to the concern for propagation of HIV *in vitro* during manufacturing, all T cell products from HIV seropositive blood samples intended for clinical uses were grown in the presence of anti-retroviral drugs. The resultant HIV-specific T cells (HXTCs), a mixture of CD8^+^ and CD4^+^ T cells, were able to lyse peptide-pulsed targets and suppress the viral spread of an HIV lab strain *in vitro*. Moreover, HXTCs were also capable of controlling the outgrowth of autologous reservoir-derived HIV and, more importantly, clearing resting CD4^+^ T cell reservoir post LRA treatment *in vitro* (Sung et al., 2015). To further increase the ability of infused T cells to target HIV escape variants, a bivalent mosaic of peptides targeting the regions of Gag and Pol antigens known to be functionally conserved, common in escape variants, and associated with natural immune protection (Ondondo et al., 2016) along with the Nef peptide library were used to manufacture T cell products using a similar platform as HXTCs (Patel et al., 2020). These HIV-specific T cells targeting non-escape epitopes (HST-NEETs) demonstrated peptide specificity, but their ability to control viral spread remains to be determined. While a significant proportion of HXTCs or HST-NEETs were likely derived from pre-existing memory T cells in HIV-infected individuals, they can also be generated from seronegative adult or cord blood donors, broadening its applicability in settings such as post-HSCT treatment or third-party VST banks (Patel et al., 2016, 2018, 2020).

The *in vivo* efficacy of HXTCs has been evaluated in a phase 1 clinical trial (NCT02208167). When infused to HIV-infected participants on ART, HXTCs were safe and well-tolerated, but showed little impact on the frequency of cells harboring replication competent HIV, measured by the quantitative viral outgrowth assay (Sung et al., 2018). An ongoing clinical study (NCT03485963) was initiated to test HST-NEETs in ART-suppressed, HIV-infected individuals. While preliminary results suggest that these HIV-specific T cell products are safe, feasible to manufacture, and functional in *in vitro* assays, they were not expected to demonstrate therapeutic effects for several reasons. First, the viral antigen levels in ART-suppressed individuals are likely too low to trigger the activation of multi-specific T cells. Second, the infusion products contain CD4^+^ T cells, which can benefit the accompanying CD8^+^ T cells but also become targets of HIV infection. Lastly, during the manufacturing process, cell products may acquire the expression of co-inhibitory receptors and/or lose the expression of proper homing receptors, resulting in poor persistence and homing ability to the latent reservoir.

## Providing *In vivo* Help to Infused T Cells

Strategies have been proposed to assist infused T cells to overcome the immunosuppressive mechanisms deployed by HIV. To increase viral antigen levels for detection by TCRs or CARs, administration of LRAs during ART can potentially reactivate viral replication without causing massive viral rebound as proposed in the “shock and kill” or “kick and kill” approach (Archin et al., 2012b; Deeks, 2012; Lopez, 2013). Although the potency and durability of LRAs on increasing immunogenicity of the viral reservoir and their immunomodulatory effects on infused T cells *in vivo* remain unclear (Spivak and Planelles, 2018), combining LRAs with ACT could in theory reduce the size of the latent reservoir as supported by *in vitro* studies using viral outgrowth assays (Shan et al., 2012; Sung et al., 2015). An ongoing clinical trial is testing the safety and efficacy of a broadly-neutralizing antibody (bNAb)-based CAR-T cell therapy combined with a histone deacetylase inhibitor (HDACi), Chidamide, in ART-suppressed patients without treatment interruption (NCT03980691). Another phase 1 trial is evaluating the effects of HXTCs and another HDACi, Vorinostat, on ART-suppressed participants who show increased cell-associated viral RNAs in response to the initial Vorinostat administration (NCT03212989). These clinical studies will provide valuable information about the feasibility of using LRAs as adjuvants to boost the adoptive T cell responses without the need to interrupt ART.

One of the biggest concerns of using T cells to treat HIV is the risk of infection of the infused CD4^+^ T-cell compartment by the reactivated virus. One option is to prevent HIV entry by using allogeneic T cells from donors with the homozygous CCR5 Δ32 mutation, but this approach is not applicable to most HIV-infected individuals. Alternative strategies to confer HIV resistance include genome-editing technologies. A clinical study reported that the infusion of autologous CD4^+^ T cells edited by zinc finger nuclease (ZFN) to delete the *CCR5* gene was largely safe, and that the gene-modified cells engrafted and persisted (NCT00842634 Tebas et al., 2014). In a case report where an HIV-infected patient with acute lymphoblastic leukemia received an allo-HSCT with CD34^+^ hematopoietic stem and progenitor cells edited by clustered regularly interspaced short palindromic repeats technology to delete the *CCR5* gene, full chimerism was achieved and the resultant *CCR5*-ablated CD4^+^ T cells persisted for months (NCT03164135 Xu et al., 2019). HIV fusion inhibitors such C34 and C46 have also been used to prevent infused T cells from HIV infection in clinical trials, but the results have not been reported (NCT03020524 and NCT01734850). An ongoing clinical trial is examining the efficacy of infusing autologous T cells modified to deactivate the *CCR5* gene and to express a CD4-based CAR, followed by ART interruption (NCT03617198). Moreover, autologous CD4^+^ T cells transduced with a lentiviral vector expressing an antisense targeting HIV *env* were infused to 13 ART-treated patients followed by treatment interruption, attempting to block viral replication upon infection (Tebas et al., 2013). Strategies to confer HIV resistance should be considered for any types of cell-based therapy to avoid exacerbation of viral rebound.

Obtaining clinically relevant numbers of T cells for infusion often requires multiple rounds of TCR stimulation, which can result in an effector memory (T_EM_)-enriched cell population lacking CD62L and CCR7 expression (Sung et al., 2018; Patel et al., 2020). T_EM_ cells have been shown to demonstrate inferior persistence and anti-tumor function *in vivo* compared to T stem cell memory (T_SCM_) or central memory (T_CM_) cells (Berger et al., 2008; Gattinoni et al., 2011). Not only is the expression of the homing receptors–CD62L and CCR7–linked to a gene signature favoring long-term persistence and better effector function (Klebanoff et al., 2005), but they are also required for T cell trafficking to secondary lymphoid tissues (Weninger et al., 2001), the major site of the viral reservoir (Boritz and Douek, 2017; Dimopoulos et al., 2017). Additional homing receptors, such as α4β7 and CXCR5, are also crucial for T cells to traffic to gut-associated lymphoid tissues and B cell follicles, respectively (Schaerli et al., 2000; Arthos et al., 2018). Ensuring the expression of these homing receptors, either by gene-engineering approaches or improved culture protocols, may allow efficient accumulation of infused T cells in the viral reservoir. Furthermore, co-inhibitory receptors such as CTLA-4, PD-1, and Tim-3 are induced transiently following TCR stimulation, and remain expressed on exhausted T cells (Kuchroo et al., 2014). Blockade of these pathways *in vivo* by administration of monoclonal antibodies, namely checkpoint inhibitors, has achieved great clinical benefits in the cancer immunotherapy field (Korman et al., 2006; Wei et al., 2018). Ipilimumab, a fully humanized monoclonal antibody targeting CTLA-4 shown to improve the overall survival of patients with metastatic melanoma (Hodi et al., 2010), was used to treat HIV-infected participants resistant to ART (Colston et al., 2018). Ipilimumab was overall well-tolerated but showed little clinical efficacy. Combining checkpoint inhibitors with cell therapies may be an attractive strategy, but potential immune-related adverse effects must be carefully evaluated. Blocking the inhibitory pathways may provide a boost to infused T cells, but it may concurrently cause autoimmunity or awaken the quiescent latent reservoir, as increased viral loads had been observed in nearly 60% of the participants receiving ipilimumab in a prior trial (Colston et al., 2018).

## Summary and Future Perspective

The two cases of long-term viral remission without ART may not offer a generalized therapeutic approach for a functional cure, but they have undoubtedly provided valuable insights into the underlying mechanisms and inspired novel research toward the ultimate goal. Modern ART regimens have redefined HIV diagnosis from a death sentence to a chronic disease, and their ability to stop active viral replication may give immunotherapy an opportunity to eradicate the virus altogether. Given the immune evasion mechanisms shared by cancer cells and HIV (Mylvaganam et al., 2019), researchers in the HIV field are hoping to mirror the success enjoyed by cancer immunotherapy experts. The fact that ART-treated, HIV-infected individuals have a higher risk of malignancies further tangles the two disease types and creates more treatment options for HIV.

**Figure 1:**
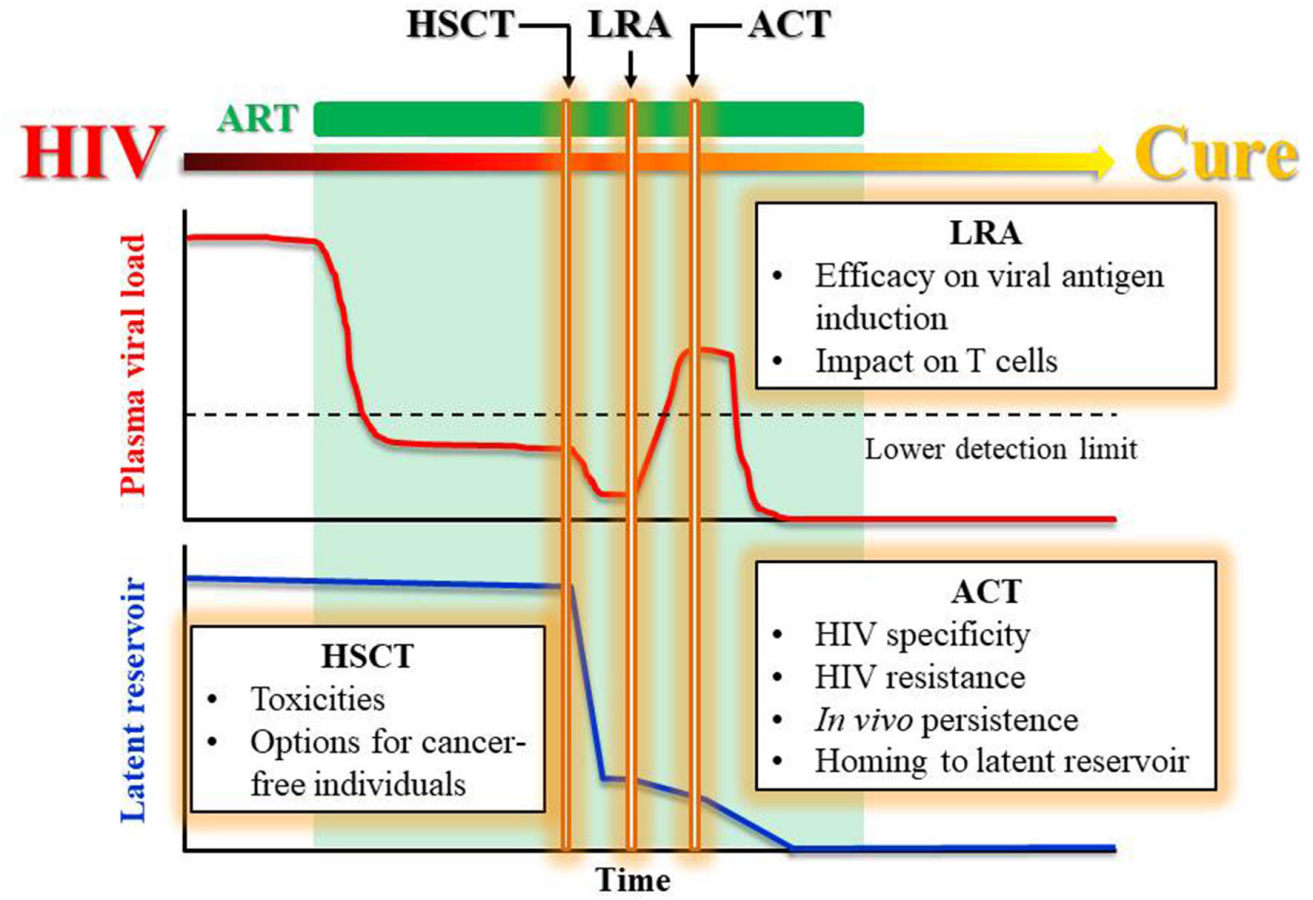
Proposed treatments leading to a functional cure for HIV. Proposed treatment plan to achieve a functional HIV cure by a stepwise reduction in viral load and latent reservoir while maintaining ART. The rectangle boxes list the challenges faced by each therapeutic component including HSCT, LRA, and ACT.

Replacing the HIV-harboring immune system with an HIV-resistant immune system seems to be essential in curing HIV. The chemo-radiotherapy preparative regimen together with the allodepletion of the recipient's immune system should remove most of the latent reservoir. However, the delayed HIV rebound observed in the Boston patients and Mississippi baby (Ananworanich and Robb, 2014) months after ART interruption indicates that the HIV still present in the reservoir can re-infect the host T cells before HIV-specific immunity can be elicited *in vivo*. Nevertheless, even allo-HSCT with HIV-resistant donors did not cure most patients, raising the question: how was the residual reservoir post HSCT eradicated or contained in those patients who did achieve a cure? While we cannot rule out the possibility that the reactivated virus unable to re-infect a donor cell eventually died off, it is more likely that the cure was achieved only in the cases where the donor immune system eradicated the reservoir via a graft-vs.-HIV reservoir response. Therefore, we believe that, in the post-HSCT settings, infusion of HIV-specific, HIV-resistant T cells with the help from continued ART to suppress new infection and the help from LRAs to reverse viral latency can be a route to a functional cure (Figure 1).

In cancer-free HIV-infected individuals, however, efficient and safe alternatives to HSCT are needed to initiate a reduction in the viral reservoir before ACT takes effect. It remains to be seen if the combination of HIV-specific T cells and LRAs can exert any clinical benefits in ART-suppressed patients (NCT03212989), but the infused T cells may be at disadvantages for three reasons: (1) the latent reservoir is expected to be substantially bigger without lymphodepletion, (2) the lymphoreplete host environment is suboptimal for the expansion of infused T cells, and (3) the infused T cells may be vulnerable to HIV infection upon viral reactivation. The occurrence of “elite controller” individuals who can keep the virus in check without ART by maintaining strong HIV-specific T cell responses (Saez-Cirion et al., 2007) support the idea of harnessing the immune system to achieve a functional cure for a broader population. Although CD8^+^ T cells appear to be the main effectors to kill the virus, other genetic and cellular components may also be required for “elite” anti-HIV immunity (Walker and Yu, 2013). A multifaceted approach will therefore be needed to eliminate the HIV reservoir and provide better curative options to patients.

## Author Contributions

P-HL wrote the manuscript. MK, PH, and CB reviewed and edited the manuscript. All authors contributed to the article and approved the submitted version.

## Conflict of Interest

MK serves on an advisor board for Gilead Sciences. PH is a co-founder and serves on the board of directors of Mana Therapeutics. CB serves on an advisory board for Cellectis, serves on the board of directors for Cabaletta Bio and is a co-founder of Mana Therapeutics and Catamaran Bio and serves on their scientific advisory board. In addition she has stock ownership in Neximmune and Torque Therapeutics. The remaining author declares that the research was conducted in the absence of any commercial or financial relationships that could be construed as a potential conflict of interest.
